# Tool for automatic macrozone characterization from EBSD data sets of titanium alloys

**DOI:** 10.1107/S1600576723003862

**Published:** 2023-05-23

**Authors:** B. Fernández Silva, M. Jackson, K. Fox, B. P. Wynne

**Affiliations:** a Department of Material Science and Engineering, University of Sheffield, Sheffield S1 3JD, United Kingdom; b Rolls-Royce plc, PO Box 31, Derby DE24 8BJ, United Kingdom; c Department of Mechanical and Aerospace Engineering, University of Strathclyde, 75 Montrose Street, Glasgow G1 1XJ, United Kingdom; Ecole National Supérieure des Mines, Saint-Etienne, France

**Keywords:** macrozones, electron backscatter diffraction, titanium alloys, texture, characterization, crystal misorientation criteria

## Abstract

Microtexture heterogeneities are commonly found in titanium forgings because of the thermomechanical processing. Also known as macrozones, these regions can reach millimetres in length, with grains sharing a similar crystallographic orientation leading to less resistance to crack propagation. Since the link between macrozones and the reduction of cold-dwell-fatigue performance on rotative components in gas turbine engines was established, efforts have been put into macrozone definition and characterization. The electron backscatter diffraction (EBSD) technique, widely used for texture analysis, allows for a qualitative macrozone characterization; however, further processing is required to define the boundaries and disorientation spread of each macrozone. Current approaches often use *c*-axis misorientation criteria, but this can sometimes lead to a large disorientation spread within a macrozone. This article describes the development and application of a computational tool implemented in MATLAB for automatic macrozone identification from EBSD data sets on the basis of a more conservative approach where both the *c*-axis tilting and rotation are considered. The tool allows for detection of macrozones according to the disorientation angle and density-fraction criteria. The clustering efficiency is validated by pole-figure plots, and the effects of the key parameters defining the macrozone clustering (disorientation and fraction) are discussed. In addition, this tool was successfully applied to both fully equiaxed and bimodal microstructures of titanium forgings.

## Abstract 2

An algorithm for macrozone characterization in titanium alloys based on crystal misorientation criteria is presented and applied to electron backscatter diffraction (EBSD) data sets of titanium forgings.

## 1. Introduction

Near-α and α+β titanium alloys are used for demanding applications in gas turbine jet engines. Commonly manufactured by thermomechanical processing, titanium mill products are known to have localized microtexture regions (MTRs) of similar crystallographic orientation, also called macrozones. These regions were first revealed in the 1990s by Woodfield *et al.* (1995 ▸), whose research linked the presence of macrozones to cold dwell fatigue (Hinson, 1995 ▸; Garvey, 2000 ▸). Cold-dwell-fatigue susceptibility of titanium alloys for the aerospace industry has been a matter of investigation since the 1970s (Bache, 2003 ▸; Evans & Gostelow, 1979 ▸; Song & Hoeppner, 1989 ▸). Since then, a vast amount of effort has been put into investigating macrozone origin and evolution in titanium alloys during thermomechanical processing (Germain *et al.*, 2008 ▸; Gey *et al.*, 2012 ▸; Pilchak *et al.*, 2013 ▸; Venkatesh *et al.*, 2016 ▸), and their influence on the dwell-fatigue performance of Ti834 (Bache *et al.*, 1997 ▸; Uta *et al.*, 2009 ▸), Ti6242 (Germain *et al.*, 2020 ▸; Sinha *et al.*, 2006*b* ▸) and Ti-6Al-4V (Le Biavant *et al.*, 2002 ▸; Lavogiez *et al.*, 2020 ▸; Stubbington & Pearson, 1978 ▸; BEA, 2017 ▸), hence the need to establish a methodology for macrozone characterization.

### 1.1. Macrozone formation

Macrozones are believed to be a result of thermomechanical processing in the β and α+β regions where large colonies are generated by phase transformation (β → α) and variant selection, leading to regions with preferential selection of orientations (Germain *et al.*, 2005*b* ▸; Stanford & Bate, 2004 ▸). Upon cooling, the parent β grains transform into large colonies following the Burgers orientation relationship (BOR), consisting of relatively thick lamellas from which α_p_ grains are generated by deformation and globularization. Although the BOR is believed to be broken by the high levels of deformation imposed during globularization by the generation of high-angle boundaries (Weiss *et al.*, 1986 ▸), these grains tend to deform in the same way, maintaining a common *c*-axis orientation of the hexagonal close packed (h.c.p.) cell within the initial colony (Woodfield *et al.*, 1995 ▸) which leads to macrozones consisting of α_p_ grains that extend over the initial colony size or beyond (Germain *et al.*, 2005*a* ▸).

### 1.2. Macrozone definition

In previous research carried out on macrozone identification, the definition of macrozones differs if the investigation is focused on macrozone formation and evolution in titanium manufacturing or its effect on mechanical performance in the final component. When investigating macrozone formation and evolution, researchers focus on the density and extension of microstructural features sharing a common crystallographic orientation. For instance, Germain *et al.* (2005*a* ▸,*c* ▸) analysed macrozones in Timetal 834 billet material with 30% α_p_ surrounded by lamellar α_s_ colonies by electron backscatter diffraction (EBSD), where each macrozone showed a strong local texture with high variations in orientation and density of the main texture component, *i.e.* α_p_; they concluded that a macrozone can be defined as a cluster of α_p_ sharing a single texture component with 20° of spread. Davies (2009 ▸) and Uta (2009 ▸) analysed the texture variation through a cross section of Ti834 and Ti-6Al-4V billets, respectively; the macrozones were preferentially elongated in the axial direction with the *c* axis parallel or perpendicular to this direction, with misorientations up to 30° from a predominant α texture component. In summary, the disorientation between the grains within a macrozone forming a single component of the texture has been found to be between 20 and 30°. However, these disorientation criteria are based on macrozone observation in orientation maps only, with no link to mechanical behaviour. When assessing the effect of macrozones on mechanical properties, such as in dwell fatigue, the disorientation of the macrozone with resepct to the deformation axis is of interest. The orientation of the macrozone within a large volume of material changes from one region to the next, leading to neighbouring regions with different crystallographic texture. Due to the strong plastic anisotropy of the h.c.p. crystal of the α phase (Fisher & Renken, 1964 ▸) and given a loading direction (LD), macrozones are defined as soft (favourably orientated for slip) or hard (unfavourably orientated for slip) regions that, when neighbouring each other, will lead to load shedding in the soft region triggering the nucleation of cracks in the hard region caused by the generation of quasi-cleavage facets (Uta *et al.*, 2009 ▸; Sinha *et al.*, 2006*b* ▸). These facets are often observed in α_p_ grains with the basal plane at angles between 10 and 30° from the LD (Uta *et al.*, 2009 ▸; Sinha *et al.*, 2007 ▸) under dwell-fatigue loading conditions. However, different facet angle ranges have been observed experimentally between fatigue and dwell fatigue: facets near perpendicular to the LD were found for normal fatigue loading conditions, while facets showing a deviation of 10–15° with respect to the basal plane were observed for dwell loading conditions (Sinha *et al.*, 2006*a* ▸; Evans & Bache, 1994 ▸; Gosh *et al.*, 2007 ▸).

Work on crystal plasticity models by Dunne *et al.* (2007 ▸) has defined the ‘rogue pair’ as the worst combination of hard–soft orientation for quasi-cleavage facet nucleation, where a hard grain with its basal plane nearly perpendicular to the LD is neighbouring a soft grain with its prismatic plane normal at 70° to the load. Moreover, several researchers have established differences between crack initiation and propagation regions: for example, Bantounas *et al.* (2010 ▸) highlighted a difference between grains orientated for crack initiation (*c* axis < 15° from LD) and those for crack propagation (*c* axis at 15–40° from LD) under high cycle fatigue, while probabilistic work on crack plane orientations has revealed that the critical crystallographic orientations for nucleation sites are found in grains with the *c* axis between 10 and 30° from LD preferentially (Ozturk *et al.*, 2017 ▸; Liu *et al.*, 2023 ▸). More recently, research by Harr *et al.* (2021 ▸) suggests that a certain degree of connectivity between grains is required for crack growth in Ti6242 bimodal microstructures under dwell-fatigue loading conditions, and therefore both grain connectivity and orientation criteria must be considered when characterizing macrozones linked with dwell-fatigue failure. In addition, the work of Liu & Dunne (2021 ▸) has established the most damaging macrozones under dwell-fatigue conditions as being macrozones with a high aspect ratio of >∼4 elongated near normal to LD and with their *c* axes within ∼15° from LD. From previous research, it is clear that there is no agreement on how to define a macrozone. The definition of macrozone differs from a formation and evolution study to a failure and mechanistic perspective and, therefore, the ultimate definition of a macrozone is yet to be established.

### 1.3. Macrozone characterization

EBSD has been widely used in most studies on texture for macrozone analysis. Although macrozones are visually detected from the orientation maps created with EBSD data, further research needs to be carried out to fully characterize macrozones in terms of degree of spread, average misorientation and density. Within an EBSD map, macrozones can be detected in inverse pole figure (IPF) orientation maps, since the colouring key represents the orientation of the crystal *c* axis with respect to a reference axis and, therefore, macrozones that share a common *c* axis will appear in one equally coloured cluster (Davies *et al.*, 2018 ▸). Alternatives to EBSD have also been considered for macrozone characterization, such as optical cross-polarized light (Lütjering & Williams, 2007 ▸) or heat tinting (Lunt, 2014 ▸; Nasseri, 2013 ▸). Ultrasonic attenuation inspection has been successfully used in titanium alloys, where the signal obtained from the ultrasonic response could be correlated with the presence of macrozones (Humbert *et al.*, 2009 ▸). A more recent technique is based on a laser ultrasonic method, spatially resolved acoustic spectroscopy, where surface acoustic waves, whose frequency varies with the crystallographic orientations, are propagated on a surface; this technique is able to cover large areas (Li, 2012 ▸; Sharples *et al.*, 2007 ▸). However, the possibility of data post-processing in these alternative methodologies is limited. More recently, researchers have been using automatic tools to characterize macrozones from EBSD data sets, allowing the identification of macrozones according to their *c*-axis orientation (Venkatesh *et al.*, 2020 ▸, 2016 ▸; Pilchak *et al.*, 2013 ▸). The commercial software *TiZone* (Materials Resources LLC; Shaffer, 2013 ▸) identifies MTRs by clustering data points according to their orientation and spatial location; individual macrozones are identified on the basis of their *c*-axis orientation, and subsequently metrics are generated by *n*-point statistics to identify continuous regions of microtexture. The open-source software *DREAM3D* (Groeber & Jackson, 2014 ▸) allows the generation of user-defined pipelines, which can identify α_p_ grains by applying strict misorientation criteria. These criteria are then considered to group the data set into segments according to wider *c*-axis misorientation criteria. In this approach, the interconnectivity between points is not enforced since α_p_ grains might not be necessarily interconnected due to their distribution in the microstructure or the resolution of the map. The *c*-axis approach may lead to the identification of macrozones sharing a common *c* axis but whose rotation about the *c* axis is not considered. In other words, hard macrozones that are defined by a common *c* axis will have the basal planes aligned, while soft macrozones sharing a common *c* axis might show misalignments from one prismatic plane to the next due to rotation about the *c* axis, leading to a different definition of macrozones despite the same criteria having been used.

This work aims to provide support on the investigation of macrozones in titanium forgings, hence the development of a tool for automatic macrozone identification from EBSD data sets. In contrast to the other tools available, the current tool considers the full rotation of the h.c.p. crystal instead of only the *c*-axis misorientation criteria. To the authors’ knowledge, the algorithms developed in the applications previously mentioned have not been made publicly available. This algorithm was developed in MATLAB independently of the methods that are currently available for macrozone analysis. This article is structured to provide a few key equations used in the development of this tool, followed by the optimization and application of the tool in titanium alloy EBSD data sets. The code for macrozone identification is written in MATLAB, in addition to scripts that enable one to load the data in the correct format as well as macrozone pole-figure plotting. This approach may potentially suffer from the dependence on subjective choice of key input parameters by the user, so the effects of these choices are discussed. This tool has been designed for the analysis and especially post-processing of EBSD data from HKL systems.

## 2. Coordinate systems and orientation descriptors

This methodology for macrozone identification is based on the clustering of pixels with similar crystallographic orientation considering the full misorientation (*c*-axis tilting and rotation) of the crystals. For that, a series of calculations are required to obtain the orientation of each pixel in the data set, the misorientation between them and their clustering, applying average quaternions. The equations utilized in this algorithm are described in this section. A detailed explanation on how these equations are used is shown in the step-by-step procedure in Section 3.

### 2.1. Coordinate system

The most convenient way to describe the orientation of a crystal is by using two orthonormal coordinate systems: the crystal coordinate system (**C**_crystal_) and the sample coordinate system (**C**_sample_), which is generally chosen to be aligned with some well-defined process directions, *e.g.* rolling, normal and transverse directions if the material was rolled (Engler & Randle, 2009 ▸). The crystal orientation is then defined by the rotation required to make one reference frame identical to the other. This rotation can be defined by several orientation descriptors, such as Euler angles, orientation matrix, angle/axis pairs or quaternions.

### 2.2. Orientation matrix

The orientation matrix **G** describes the rotation between the two coordinate systems: 

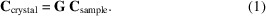

The **G** matrix is obtained by the multiplication of three rotation matrices that are a result of each passive rotation defined by each of the three Euler angles in the Bunge notation [φ_1_, Φ, φ_2_] (Rowenhorst *et al.*, 2015 ▸): 

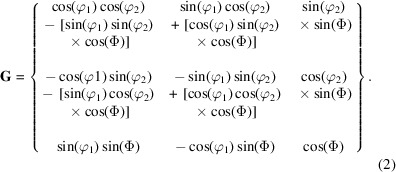

Depending on the crystal symmetry of the system, there are a number of physically indistinguishable solutions for an orientation or misorientation, known as crystallographically related solutions (Engler & Randle, 2009 ▸). These include 24 solutions for an orientation matrix of a material having cubic symmetry and 12 solutions for hexagonal symmetry (Kocks *et al.*, 1998 ▸). For a given orientation matrix (**G**), the full set of solutions (**G**_*i*_) is obtained by pre-multiplying the calculated orientation matrix by each of the symmetry matrices (**S**_*i*_) that describe the symmetry of the crystal:

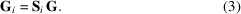

Each of the resulting matrices describe a physically indistinguishable orientation.

### 2.3. Misorientation matrix

The misorientation matrix Δ**G** is used when the rotation of interest occurs between two crystals. Given two grains with orientations **G**_a_ and **G**_b_, the Δ**G** matrix defines the rotation required to bring the orientation of the crystal coordinate system of grain A to that of grain B. In terms of the orientation of each matrix, **G**_a_ shows the rotation back from position A to the reference position (this is an inverse rotation so **G**_a_^−1^) and **G**_b_ shows the rotation to position B: 

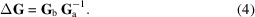

Due to the symmetry operations, there are 288 (2 × 12 × 12) and 1152 (2 × 24 × 24) crystallographically related solutions for hexagonal and cubic crystals, respectively: 

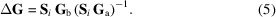

From each misorientation matrix, an axis–angle pair can be obtained consisting of the common vector to both lattices and the rotation angle to make them match (Engler & Randle, 2009 ▸). For orientation analysis, it is common to select the minimum rotation angle, known as the disorientation angle, among all axis–angle pairs (Humphreys, 2001 ▸). The angle θ is obtained from the trace of the Δ**G** matrix. When just this angle is the criterion of interest, only one set of symmetry operators must be applied: 

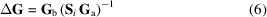

and

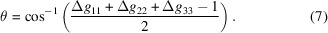




### 2.4. Quaternions

The quaternion descriptor is a vector that can be used to describe a rotation in a coordinate system. It can be obtained from the Euler angles, orientation matrix or axis–angle pairs by applying a change in variables leading to a unit quaternion, whose norm equals 1. A quaternion is represented as *q* = (*q*_0_, q) = (*q*_0_, *q*_1_, *q*_2_, *q*_3_), with *q*_0_ the scalar part and q the imaginary vector: 

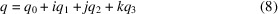

and

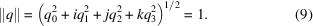

The equivalence between the Euler angles and the unit quaternion is given by (Humbert *et al.*, 1996 ▸; Morawiec & Pospiech, 1989 ▸; Rowenhorst *et al.*, 2015 ▸) 

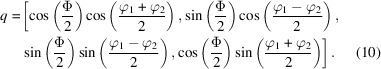

The average of a set of orientations can be computed by quaternions or orientation matrices. However, the quaternion descriptor is known to be the most accurate and efficient way to obtain an average orientation because fewer calculations are required (Humbert *et al.*, 1996 ▸): a quaternion only requires nine calculations to obtain the disorientation, while using the orientation matrices (**G** matrix) requires 27 calculations. In terms of quaternions, by using a set of orientations *q_k_* with *k* = 1,…, *n*, the average quaternion 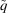 is obtained by the sum of each quaternion divided by the norm of the sum: 

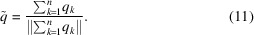




## 3. Materials and methods

**Figure 1:**
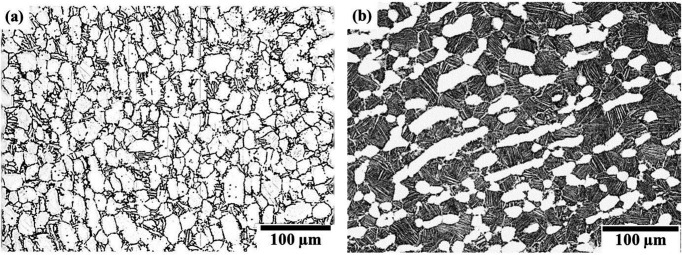
(*a*) Fully equiaxed and (*b*) bimodal microstructure of titanium forgings utilized in this work.

EBSD data sets were collected from Ti-6Al-4V and Ti834 forgings including different volume fractions of α_p_ grains. Fully equiaxed [Fig. 1 ▸(*a*)] and bimodal microstructures with a volume fraction of α_p_ grains of 25% [Fig. 1 ▸(*b*)] and an average grain size of 25 µm were utilized during the development and optimization of this tool (see Table 1 ▸). The data sets used in this work were obtained from different scanning electron microscopes (SEMs), all equipped with a Symmetry EBSD detector. *AZTEC HKL* (Oxford Instruments) was used for data acquisition and *Channel 5* (Oxford Instruments) was used for data clean-up. These data sets cover areas of 10 × 5 mm and 4 × 2 mm using step sizes of 10 and 5 µm, respectively.

**Figure 2:**
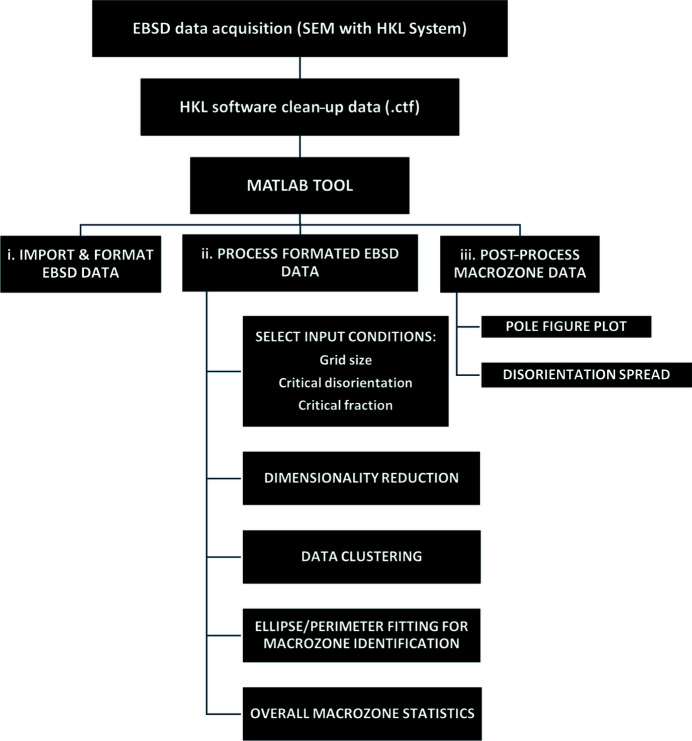
A schematic chart describing the main steps for macrozone identification.

Fig. 2 ▸ shows schematically the step-by-step procedure from orientation data acquisition to the identification of the macrozone and post-processing. For the identification of macrozones, orientation data (.ctf) acquired by EBSD with the *HKL* software, and data clean-up with *Channel 5* if required, are used to obtain data sets with 90–100% indexing. The EBSD data are loaded into a formatted data set version [Fig. 2 ▸(i)] containing the orientation matrix for each point in the data set with respect to the sample reference frame [equation (2)]. These new formatted EBSD data are then loaded into the tool for macrozone identification [Fig. 2 ▸ (ii)], for which three input parameters are selected in advance by the user:

(1) The grid size. The gird size is a kernel used for scanning the map with a step equal to the kernel size. The grid size was selected to be twice the size of the average grain size. In the current work, the grain diameter is ∼25 µm; therefore, areas of at least 50 × 50 µm were suggested, which led to the grid-size parameter being a function of the step size of the EBSD data set. The EBSD data sets used in the current investigation were obtained with step sizes of 5 and 10 µm; therefore, the grid sizes utilized for each data set are defined as 11 × 11 and 5 × 5, respectively, in order to cover at least the minimum area suggested (50 × 50 µm) to ensure that more than one single grain is used for the calculations. The grid must be defined by an odd number, as a central point is required for the misorientation calculations with the neighbouring pixels.

(2) The critical disorientation. The disorientation angle was described in equation (7) and it defines the minimum rotation between points. The critical-disorientation value defines the maximum disorientation angle allowed between pixels within a macrozone. A 20° angle is suggested as the critical-disorientation angle as it has been widely used to define the disorientation angle of features within a macrozone (Germain *et al.*, 2005*a* ▸,*c* ▸). In each kernel calculation, the misorientation is calculated between the central point and the surrounding points within the grid.

(3) The critical fraction. This parameter defines the number of points within the grid that must meet the critical-disorientation condition to be selected as part of a macrozone; therefore, it can be considered a density criterion. The critical fraction is obtained as indicated in equation (12):

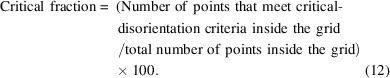

The higher the critical fraction, the higher the density of the macrozone, but fewer and smaller macrozones will be selected since it is the most restrictive condition.

To ensure accuracy, all these three parameters must be kept constant when comparing data sets. The current tool has been optimized and validated for the values shown in Table 2 ▸.

The algorithm utilizes a two-step procedure: (1) a dimensionality reduction step, where each point in the data set is either highlighted or removed on the basis of misorientation and density criteria with respect to its neighbouring data points within a user-defined area (the grid size) [equations (6) and (7)] taking the central pixel as a reference; and (2) a step for data clustering that considers only the points previously selected in the dimensionality reduction step and identifies the neighbouring points of similar orientation or within the disorientation criteria previously established, subsequently defining the macrozones. In contrast to the first step, the misorientation calculation takes place between the points of a grid and the average orientation of this grid, instead of the central point. The points that have met the criteria in this first grid are then clustered and each point will be individually used as central points in subsequent grids for scanning the map. A new average orientation matrix is calculated and used for the next misorientation calculations every time new pixels are added to the cluster. This creates a moving average that is used to cluster points from each grid until no more points meet the disorientation and fraction conditions, therefore completing the macrozone. This step is repeated until all pixels previously filtered in the first step are assigned to a macrozone or dismissed.

In contrast to the approaches previously mentioned, this tool not only considers the orientation/tilting of the *c* axis but also considers the rotation about the *c* axis, as well as resulting in more restrictive criteria. Like *DREAM3D*, no interconnectivity between pixels is enforced, but areas defined by the user are considered for the calculations. These include the density criteria, meaning that a minimum number of points must meet the misorientation criteria within an enclosed area but they do not necessarily need to be interconnected. Additionally, average quaternions are used in the second step to avoid disorientation spread within the macrozones [equations (10) and (11)]. Size and geometry statistics are then obtained for each macrozone by fitting perimeters and ellipses using scripts available online (Brown, 2022 ▸; Fitzgibbon *et al.*, 1996 ▸).

## 4. Results

**Figure 3:**
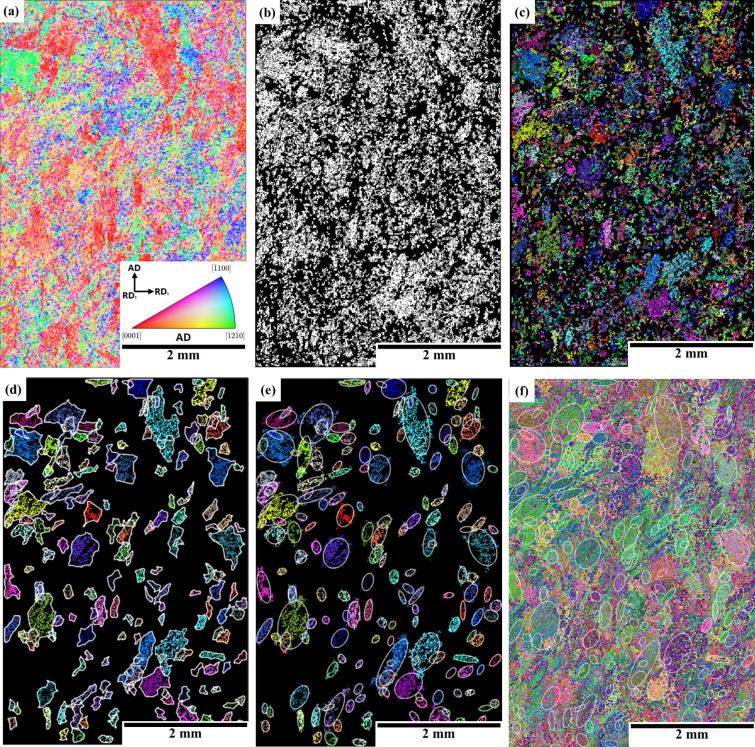
Outputs of the tool after macrozone identification in a Ti-6Al-4V billet data set (Billet 3 data set) covering an area of 4 × 6 mm with 10 µm step size, with conditions of grid/disorientation/fraction equal to 5/20/30. (*a*) The IPF orientation map with respect to AD, which is the long billet axis. (*b*) Disorientation and fraction criterion filtering. (*c*) Disorientation and clustering. (*d*) Macrozones with fitted perimeters after size threshold. (*e*) Macrozones with fitted ellipses after size threshold. (*f*) The Euler orientation map with ellipses overlapped.

An example of the application of this tool in a titanium billet specimen is shown in this section. The macrozones from a Ti-6Al-4V billet data set (Billet 3 data set from Table 1 ▸) with a fully equiaxed microstructure were identified with the current tool using the following input parameters: grid size = 5 × 5, critical-disorientation = 20° and critical fraction = 30%. Fig. 3 ▸ shows the IPF orientation map [Fig. 3 ▸(*a*)] and the outputs of several steps throughout the identification of macrozones. Fig. 3 ▸(*b*) shows a binary image after applying the first misorientation and density criteria to the data set, while Fig. 3 ▸(*c*) shows the clusters that have been identified as macrozones coloured individually. A threshold macrozone area of 10 000 µm^2^ is applied, and only macrozones above that threshold are shown in Fig. 3 ▸(*d*). Figs. 3 ▸(*d*) and 3 ▸(*e*) show the overlapped fitted perimeters and ellipses utilized to obtain macrozone-size statistical data. The ellipses are overlapped in the Euler map in Fig. 3 ▸(*f*).

Table 3 ▸ shows the results of the macrozone analysis. The area is obtained from the perimeter fitted around each macrozone [Fig. 3 ▸(*d*)]. This area is used to obtain the equivalent diameter of a circle for each macrozone. The density of the macrozone indicates the number of points inside the perimeter that are part of the macrozone, while MTR indicates how much of the area analysed is covered by macrozones. Finally, the length of the major axis is obtained from the larger axis of the fitted ellipses.

**Figure 4:**
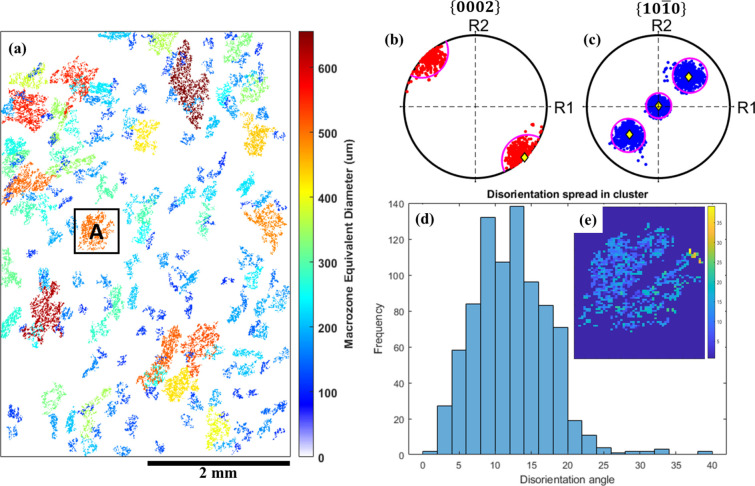
Post-processing on macrozone analysis including (*a*) macrozone clusters coloured by equivalent diameter size, (*b*) basal {0002} and (*c*) prismatic 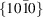 pole-figure plots (in yellow is the mean orientation of the macrozone, and the pink circle shows 20° disorientation from the mean), and (*d*), (*e*) the disorientation spread distribution of macrozone A.

Examples of data post-processing after identifying macrozones are shown in Fig. 4 ▸. Macrozones are plotted regarding their equivalent diameter dimension in Fig. 4 ▸(*a*). This is for macrozones with an area bigger than 10 000 µm^2^ as this limit was defined as the macrozone threshold size. The basal {0002} and prismatic 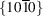 pole figures of macrozone A are displayed in Figs. 4 ▸(*b*) and 4 ▸(*c*), respectively, showing in yellow the mean orientation of the macrozone and in pink the limit of 20° disorientation from the mean. Most points in the pole figures are clustered within the 20° disorientation from the mean, and only a few points have a disorientation angle above 20°, as shown in the disorientation-angle distribution in Fig. 4 ▸(*d*). This example indicates the successful results of the code in clustering points according to the disorientation-angle criteria. The outliers, *i.e.* points above the 20° threshold, might be a result of the moving average at the second stage of the algorithm when clustering the points regarding the mean orientation of the macrozone, since this value changes slightly each time a new set of points is added to the cluster. Therefore, although most of the points are within the 20° disorientation criterion from the overall mean orientation of the macrozone, a minor number of points might not be. These occurrences were mainly observed at exterior locations of the macrozones [see Fig. 4 ▸(*e*)].

## 5. Discussion

### 5.1. Limitations and validation

**Figure 5:**
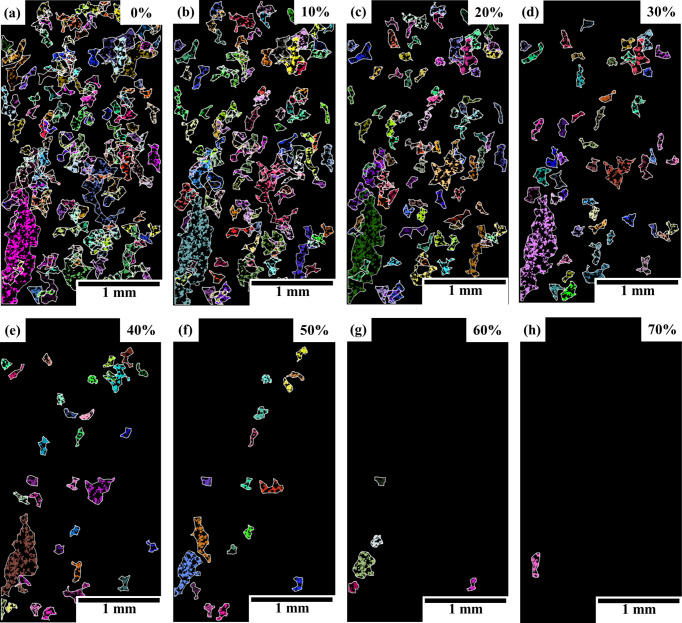
Macrozone maps with overlapping perimeters from an EBSD data set of a bimodal Ti834 (Pancake 1 data set) microstructure processed by the macrozone tool under different critical-fraction criteria from (*a*) 0% to (*h*) 70%.

The identification of macrozones by the current tool may suffer from the dependence on a subjective choice of the key input parameters by the user. The grid size and the critical-disorientation criteria are defined by the microstructural features and the disorientation of interest that defines a macrozone, respectively. However, the critical-fraction criteria significantly affect the size and density of the macrozones. Fig. 5 ▸ shows the results from the same EBSD data set (Pancake 1 data set from Table 1 ▸) under different critical-fraction values, and Table 4 ▸ summarizes the results from the macrozone analysis. As the critical fraction increases, the number and size of the macrozones drastically decrease. This is because (1) with a high critical-fraction criterion the most external points of the original macrozone will be rejected since part of the data points within the grid will not be considered macrozone points, and because (2) a large but low density macrozone will be divided into smaller but denser macrozones as the critical-fraction threshold increases. The latter effect can be observed in the bottom-left brown macrozone in Fig. 5 ▸(*e*), which has been split up into the smaller light-blue and orange macrozones in Fig. 5 ▸(*f*). By reducing the critical-fraction criterion, it is more likely that edge points will be included; however, too low a criterion would include all points, allowing for the detection of disperse macrozones (points with low connectivity within the macrozone). In this data set, at a critical fraction of 0% there were 157 macrozones; there were also 53 at 30% and only two macrozones at 70%. The maximum equivalent diameter size decreased by 11% when increasing the critical-fraction criteria to 30%, and there was a 76% reduction in size for the highest critical-fraction value investigated. However, the mean diameter only varied by less than 1% and up to 11.5% for the same critical-fraction values, respectively, while the density of the macrozones has been almost doubled (Table 4 ▸).

**Figure 6:**
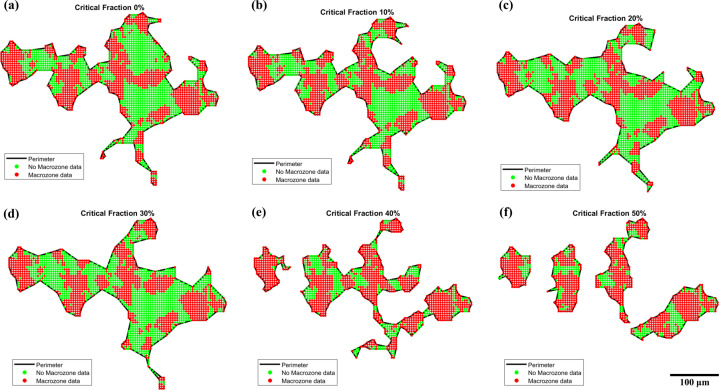
Macrozone-clustering evolution showing the perimeter, and macrozone points and non-macrozone points inside the perimeter, detected at critical-fraction values of (*a*) 0%, (*b*) 10%, (*c*) 20%, (*d*) 30%, (*e*) 40% and (*f*) 50%.

Fig. 6 ▸ and Table 5 ▸ show the analysis performed on one macrozone obtained under different critical-fraction criteria. Between 0 and 30%, the density of the macrozone remains similar despite the differences in the perimeter shape [Figs. 6 ▸(*a*)–6 ▸(*c*)], as does the number of points within the perimeter that belong to the macrozone, which, as shown in Table 5 ▸, is ∼50%. From a critical-fraction value of 40%, the macrozone breaks up into smaller and denser macrozones [Figs. 6 ▸(*e*) and 6 ▸(*f*)]. The 30% criterion [Fig. 6 ▸(*d*)] shows a closer perimeter to the real macrozone point distribution, since some of the ‘spikes’ observed at lower criteria caused by small clusters of a few pixels are rejected. Therefore, the bigger red clusters that appear to be part of the same macrozone might be individual or small groups of α_p_ grains and α_s_ colonies sharing a common orientation within the critical-disorientation criterion. Overall, as the critical-fraction criterion increases, it becomes more restrictive, and only highly dense and interconnected or closer features are detected as macrozones.

**Figure 7:**
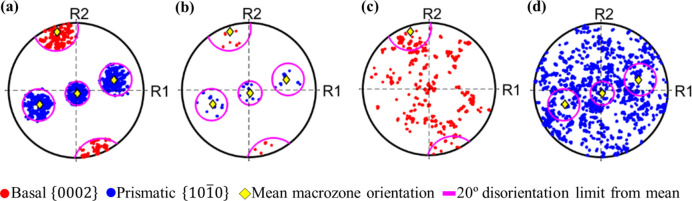
Pole-figure plots from the macrozone of Fig. 6 ▸(*d*) including (*a*) basal and prismatic poles of macrozone data, (*b*) basal and prismatic poles of selected non-macrozone data that meet the disorientation criteria, and (*c*) basal and (*d*) prismatic poles of non-macrozone data that do not meet the disorientation criteria. Basal and prismatic poles are plotted individually in (*c*) and (*d*) due to the high density of points.

**Figure 8:**
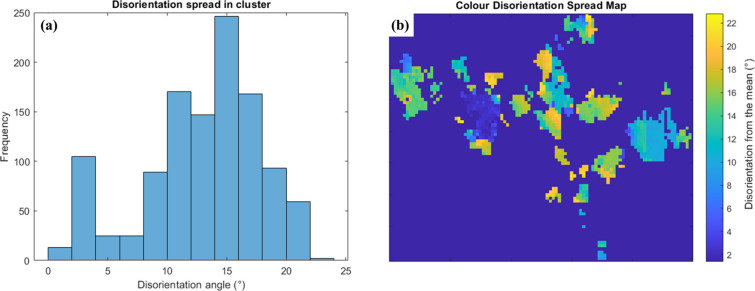
(*a*) The disorientation distribution of the macrozone of Fig. 6 ▸(*d*) and (*b*) a coloured map of the disorientation spread from the mean within the same macrozone.

Pole-figure plots showing the basal and prismatic orientations of the macrozone obtained with a 30% critical fraction are displayed in Fig. 7 ▸. Fig. 7 ▸(*a*) shows, in the same stereographic projection, the basal and prismatic poles of the points that are part of the macrozone [red points in Fig. 6 ▸(*d*)]. All the points are within 20° from the mean orientation of the macrozone, suggesting that the tool is accurately allocating the points that are within the defined criteria. This is also confirmed by the disorientation distribution shown in Fig. 8 ▸(*a*), where only ∼50 points were less than 5° from the 20° disorientation from the mean. The distribution of these points within the macrozone geometry is shown by the coloured map in Fig. 8 ▸(*b*). However, from the pole figures of the non-macrozone data, some points that meet the disorientation criteria were not selected as macrozone points [Fig. 7 ▸(*b*)]. This might be due to the critical-fraction criterion still being relatively low (30%), so that isolated points belonging to small α_p_ or α_s_ clusters that are inside the perimeter, or points that are at the very edge, are rejected. However, these pixels are less than 3% of the total macrozone points, in both cases; therefore, they will not affect the final macrozone density. The remaining non-macrozone points within the perimeter are plotted in basal and prismatic pole figures individually for clarity due to the high number of points and their spread [Figs. 7 ▸(*c*) and 7 ▸(*d*)]. In both cases there are points inside the pink circles delimiting 20° from the mean orientation. This is accounted for by a slight rotation about the *c* axis (noticed at the prismatic poles) and/or a slight inclination of the *c* axis (noticed at the basal poles) that together amount to disorientations from the mean of more than 20°. Therefore, these points no longer meet the disorientation criteria established while their basal or prismatic planes might still fall within the 20° limit from the mean.

**Figure 9:**
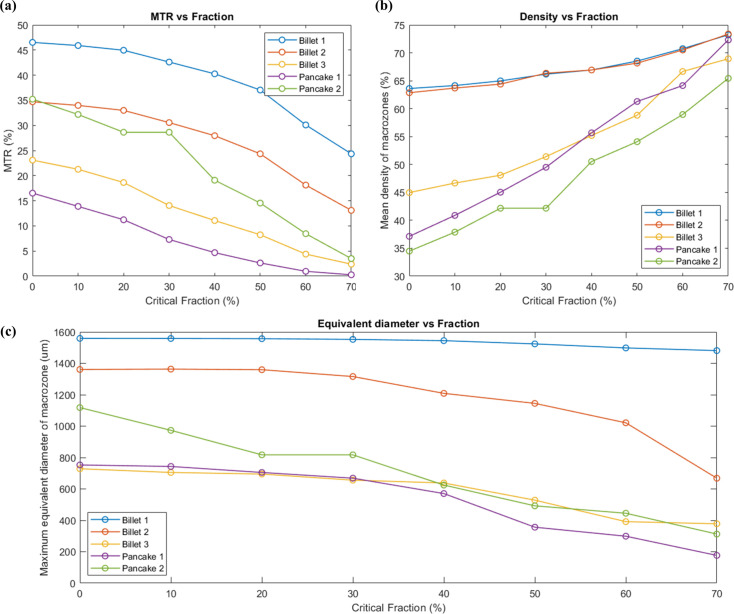
Results from the effect of the critical-fraction criteria in five different data sets on (*a*) MTR, (*b*) mean density and (*c*) maximum equivalent diameter of the macrozone.

All of the data sets shown in Table 1 ▸ were investigated to study the effect of the critical-fraction parameter. Fig. 9 ▸ shows a comparison between them in terms of MTR, density and equivalent macrozone diameter. Overall, as the critical fraction increases from 0 to 70%, there is a decrease of ∼15–20% in the area covered by macrozones per data set, MTR [Fig. 9 ▸(*a*)], while the mean density of the macrozones steadily increases with critical fraction. Such an increment is more noticeable for data sets with low-density macrozones when using a critical fraction of 0%. In Fig. 9 ▸(*b*), the data sets from Billet 1 and Billet 2 showed highly dense macrozones (close to 65%), even for lower critical-fraction values, while this is not the case for the other data sets whose starting densities of 35–45% have been almost doubled. In most cases, the maximum equivalent diameter has been relatively unaffected until a critical-fraction value of 30% [Fig. 9 ▸(*c*)], except for the Pancake 2 data set in which the diameter size decreases as the critical fraction increases from the early stages.

### 5.2. Comparison with another tool

**Figure 10:**
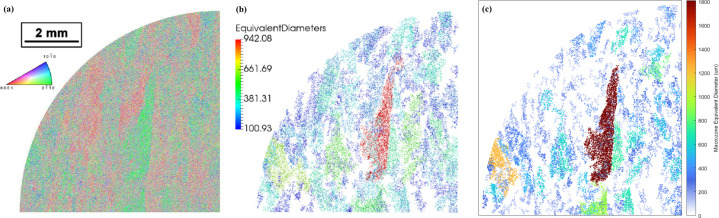
(*a*) An orientation data set obtained from Pilchak *et al.* (2016*a* ▸,*b* ▸), and macrozones coloured by circle equivalent diameters obtained using (*b*) *DREAM3D* and (*c*) the current tool in this work with conditions of grid/disorientation/fraction of 11/20/30.

The tool developed in this work was compared with the currently available open-access tool *DREAM3D* using available EBSD data sets discussed in the work of Pilchak *et al.* (2016*b* ▸). Despite the different approaches used by the tools, the aim is to contrast and compare the capabilities of the current tool to find clusters of similarly orientated points within an EBSD data set that was processed by a different approach. For this, the data set named ‘Forging Radial Face 2’ from the NIST repository (Pilchak *et al.*, 2016*a* ▸) was used. Fig. 10 ▸ shows the orientation map and the results from *DREAM3D* for the equivalent diameter obtained using a disorientation criterion of 20° compared with the results of the current tool using the same disorientation criteria [Fig. 10 ▸(*c*)]. Table 6 ▸ shows the results from both tools in terms of equivalent diameter, length of the major axis and number of macrozones detected. Both the images and the macrozone statistical results from the *DREAM3D* software were obtained from the current results shared in the repository, and the *DREAM3D* pipeline utilized for that investigation was not used by the current authors.

The results are plotted as coloured maps according to the equivalent diameter macrozone size (Fig. 10 ▸). The lower bound dimension in the data obtained by *DREAM3D* was selected for features with semi-major (ellipse fit) axes of less than 100 µm, while the current tool uses a macrozone-size threshold of 10 000 µm^2^ (which is equivalent to a minimum major-axis value of 112 µm in this data set, as shown in Table 6 ▸). The two sets of results share some similarities in the distribution and shape of the macrozones, clearly shown by the identification of the red large macrozone in the centre of both maps [Figs. 10 ▸(*b*) and 10 ▸(*c*)]. However, the main difference is that more of the area within the data set has been rejected by the current tool, since it has not been selected as part of any macrozone (white background), while a larger number of points have been allocated to macrozones by the *DREAM3D* tool. This could be due to a slight difference in the lower bound criteria between the tools, as well as the higher restriction implied by considering the full rotation of the crystal against the *c*-axis approach. Moreover, greater differences are noticed when comparing the macrozone-size statistics between the tools (Table 6 ▸), especially for the largest macrozone highlighted in red in the coloured maps [Figs. 10 ▸(*b*) and 10 ▸(*c*)]. This macrozone has an equivalent diameter of 1806 µm obtained by the current tool compared with the 942 µm from *DREAM3D*, despite being the same feature [Figs. 10 ▸(*b*) and 10 ▸(*c*)]. As described by Pilchak *et al.* (2016*b* ▸), the size and shape of the macrozones in each two-dimensional plane were approximated to ellipses quantified using the length of their major and minor axes, similarly to the current approach to ellipse fitting. However, the results show some discrepancies regarding the length of the major axis: 1.466 mm for *DREAM3D* [Fig. 10 ▸(*b*) and Table 6 ▸] but 3.684 mm for the current tool [Fig. 10 ▸(*c*) and Table 6 ▸]. The latter value is in agreement with the length of the macrozone shown in Fig. 10 ▸ [approximately two times the scale bar in Fig. 10 ▸(*a*)]. This suggests that the methodologies to obtain the macrozone size and ellipse fitting might potentially differ between the two tools, despite their giving similar clustering results. One possible reason for this is that semi-major values of the ellipses of the macrozones have been used in *DREAM3D* by Pilchak *et al.* (2016*b* ▸) instead of the major-axis values, hence leading to a difference by a factor of two in the results compared with the current tool.

**Figure 11:**
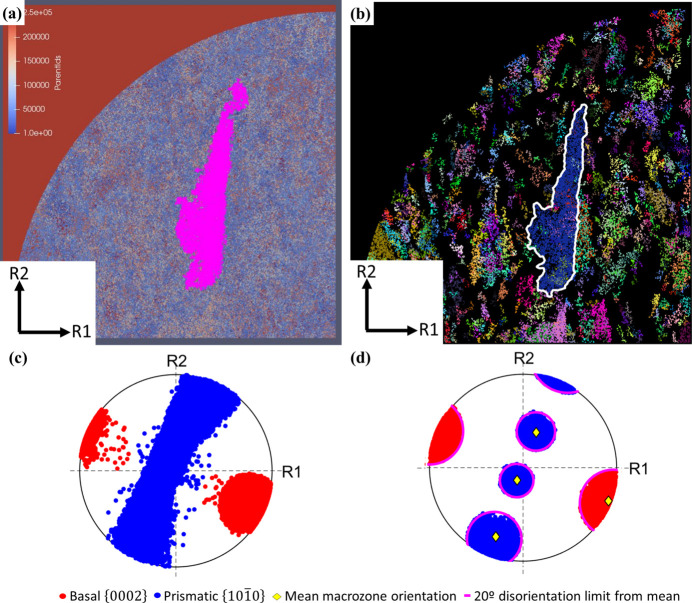
Macrozone-map outputs showing the biggest macrozone detected with the respective pole-figure plots from the data set named Forging Radial Face 2 in the NIST repository (Pilchak *et al.*, 2016*a* ▸) obtained via (*a*), (*c*) *DREAM3D* and (*b*), (*d*) the current tool. Basal and prismatic poles are plotted combined in the same pole-figure plot.

The *DREAM3D* values in Table 6 ▸ are referred to as ‘parent equivalent diameter’ and ‘parent major axis’ in the NIST repository (Pilchak *et al.*, 2016*a* ▸) outputs and belong to the Parent ID feature number 137851. Size measurements and Euler angles from this feature were obtained after post-processing the *DREAM3D* output files in the open-source software *Paraview* (Ahrens *et al.*, 2005 ▸). Pole-figure plots and colour maps highlighting the macrozones of interest obtained by *DREAM3D* and the current tool are shown in Fig. 11 ▸. Despite the similarities in the geometry of the macrozone detected by both tools [Figs. 11 ▸ (*a*) and 11 ▸(*b*)], the pole-figure plots greatly differ, showing clear differences in the orientations defined by the points within each macrozone. The *c*-axis approach shows the basal poles mainly agglomerated at ∼30° from the *R*1 reference axis, while the prismatic poles are evenly distributed along *R*2, as shown in Fig. 11 ▸(*c*), where *R*1 and *R*2 are two random orthogonal axes used for reference. The more restrictive approach of the full misorientation in the current tool results in a more defined pole figure, Fig. 11 ▸(*d*), with the basal pole similarly aligned, but in this case the prismatic poles are clearly defined within a 20° misorientation threshold. This pole figure also shows in yellow the mean orientation of the macrozone and in pink the 20° limit from the mean, which highlights the accuracy of the tool in clustering the points according to the criteria established with minimum scatter. Slightly more scatter is shown in Fig. 11 ▸(*c*).

Both tools have shown their capabilities to detect macrozones, with the main differences in the clustering approach (*c* axis versus full misorientation) and potential differences in the way that macrozone sizes are obtained. The use of one tool over the other would depend on how the user wants to define the macrozones to be identified based on the orientation spread within them, as shown in Figs. 11 ▸(*c*) and 11 ▸(*d*).

## 6. Conclusions

In the current study, a tool for post-processing EBSD orientation data has been developed for macrozone identification. It utilizes a two-step procedure based on disorientation and density criteria that relies on three key parameters. In contrast to other approaches previously mentioned, this tool not only considers the rotation about the *c* axis but also considers the tilting, resulting in more restrictive criteria. Although no interconnectivity between pixels is enforced, enclosed areas defined by the user are considered for the calculations; hence, the closeness between points can be adjusted as desired by the user. The three parameters need to be selected by the user, so the tool may potentially suffer from the dependence of a subjective choice of the key parameters (disorientation and fraction); the effects of these choices have been discussed in order to facilitate their selection. Here, a selection of values for each parameter have also been suggested, and the authors recommend that each user adjusts these parameters according to their own research interests. The measures selected are based on geometrical considerations alone and do not differentiate between ‘good’ and ‘bad’ macrozones based on average *c*-axis misorientation with LD or another direction of interest.

This tool was successfully applied to EBSD titanium forgings data sets with bimodal and fully equiaxed microstructure. The validation was performed by pole-figure plots of the macrozones, and the disorientation spread was found to be ∼5° from the disorientation criteria enforced. In addition, this tool was applied to data sets available in online repositories for which macrozones were obtained by other means. Despite the variation in the macrozone size due to a potentially different approach, especially in obtaining size statistics, the current tool has successfully identified macrozones highlighted by other tools under the same disorientation value.

Overall, in this work, we have presented a tool for research purposes that has shown the ability to detect macrozones successfully in titanium forgings. Additionally, when the key parameters for macrozone definition remain constant, this tool could potentially be implemented as a quality-control step within a titanium forgings supply chain to assess macrozones.

## 7. Data and code availability

The macrozone-tool source code, instructions and example orientation data sets used in this publication are available at https://doi.org/10.5281/zenodo.7236443. A MATLAB licence is required to use this code.

## Supplementary Material

The macrozone-tool source code, instructions and example orientation data sets: https://doi.org/10.5281/zenodo.7236443

## Figures and Tables

**Table 1:** Summary of EBSD data sets

Data sets	Alloy	Microstructure
Billet 1	Ti834	Equiaxed
Billet 2	Ti834	Equiaxed
Billet 3	Ti-6Al-4V	Equiaxed
Pancake 1	Ti834	Bimodal
Pancake 2	Ti834	Bimodal

**Table 2:** Input parameters for macrozone identification in equiaxed and bimodal microstructures

Input parameter	Values
Grid size†	5 × 5, 11 × 11
Critical disorientation	20°
Critical fraction	0–30%

† Grid size selected for step sizes of 10 and 5 µm, respectively.

**Table 3:** Summary statistics from macrozone analysis of the orientation data set in Fig. 3 ▸

Macrozone	Mean	Max	Min	Std dev.
Area (µm^2^)	40105.4	337950.8	10030.1	52719.6
Equivalent diameter (µm)	199.8	655.9	113.0	105.7
Density (%)	51.4	79.8	30.1	9.9
Length of major axis (µm)	274.4	998.8	125.2	149.6
				
MTR (%)	14			
No. of macrozones	182			

**Table 4:** Summary statistics from macrozone analysis under different critical-fraction criteria from Fig. 5 ▸

Critical fraction (%)	0	10	20	30	40	50	60	70
Density (%) mean	37.1	40.8	45.0	49.5	55.6	61.3	64.1	72.3
Equivalent diameter maximum	752.6	743.1	704.8	668.4	570.6	356.2	298.9	176.7
Equivalent diameter mean	164.9	165.8	158.8	163.3	155.5	155.5	159.2	145.9
Equivalent diameter standard deviation	66.9	65.5	65.5	72.4	75.2	58.2	61.3	38.5
MTR (%)	16.5	13.8	11.2	7.3	4.7	2.6	0.9	0.3
No. of macrozones†	157	119	95	53	33	18	6	2

† There is one macrozone behind the letter (*h*) of the image.

**Table 5:** Statistics from a single macrozone obtained under different critical-fraction criteria from the analysis performed in Fig. 6 ▸

Critical fraction (%)	0	10	20	30	40†
Perimeter (points)	2370	2188	2417	2309	1338
Non-macrozone (%)	48.9	44.4	50.4	50.5	30.5
Macrozone (%)	51.1	55.6	49.6	49.5	69.6
Density (%)	51	56	50	49	70

† Data from the biggest perimeter.

**Table 6:** A summary statistics comparison from macrozone analysis of the orientation data sets analysed by Pilchak *et al.* (2016*a* ▸,*b* ▸)

	Macrozone	Mean	Max	Min	Std Dev.
Macrozone tool	Equivalent diameter (µm)	200.3	1809.2	112.9	135.6
	Length of major axis (µm)	287.5	3684.6	112.8	226.3
	No. of macrozones	619			

*DREAM3D* †	Equivalent diameter (µm)	138.7	942.1	55.6	91.1
	Length of major axis (µm)	183.3	1466.4	100.1	129.9
	No. of macrozones	349			

† Pilchak *et al.* (2016*a* ▸,*b* ▸).
